# Inflammatory Markers and Severity of Intracerebral Hemorrhage

**DOI:** 10.7759/cureus.3529

**Published:** 2018-10-31

**Authors:** Jacob E Bernstein, Paras Savla, Fanglong Dong, Bailey Zampella, James G Wiginton, Dan E Miulli, Margaret R Wacker, Rosalinda Menoni

**Affiliations:** 1 Neurosurgery, Riverside University Health System Medical Center, Moreno Valley, USA; 2 Osteopathy, College of Osteopathic Medicine - Touro University, Vallejo, USA; 3 Clinical Research, Western University of Health Sciences, Pomona, USA

**Keywords:** vascular endothelial growth factor (vegf), tumor necrosis factor alpha (tnf alpha), homocysteine (hcy), c-reactive protein (crp), intracerebral hemorrhage (ich)

## Abstract

Background and purpose

The pathogenesis of brain injury after intracerebral hemorrhage is thought to be due to mechanical damage followed by ischemic, cytotoxic, and inflammatory changes in the underlying and surrounding tissue.In recent years, there has been a greater research interest into the various inflammatory biomarkers and growth factors that are secreted during intracerebral hemorrhage. The biomarkers investigated in this study are tumor necrosis factor alpha (TNF alpha), C-reactive protein (CRP), homocysteine (Hcy), and vascular endothelial growth factor (VEGF). The aim of this study was to further investigate the effects of these biomarkers in predicting the acute severity outcome of intracerebral hemorrhage (ICH).

Methods

We conducted a retrospective chart review of patients with spontaneous ICH with TNF alpha, CRP, VEGF, and Hcy levels drawn on admission. Forty-two patients with spontaneous ICH with at least one of the above labs were included in the study. Primary outcomes included death, Glasgow Coma Scale (GCS) on admission, early neurologic decline (END), and hemorrhage size. Secondary outcomes included GCS on discharge, ICH score, functional outcome risk stratification scale of intracerebral hemorrhage (FUNC score), change in hemorrhage size, need for surgical intervention, and length of intensive care unit (ICU) stay.

Results

Forty-two patients with spontaneous intracerebral hemorrhage (ICH) were analyzed, 12 patients (28.5%) required surgical intervention, and four patients (9.5%) died. Only low VEGF serum values were found to predict mortality. TNF alpha, CRP, Hcy, and VEGF levels in our patients with ICH were not found to predict early neurologic decline and were not correlated with GCS on admission, initial hemorrhage size, change in hemorrhage size, need for surgical intervention, ICH score, FUNC score, midline shift, and length of ICU stay. CRP and Hcy were elevated in 58% and 31% of patients tested, respectively. GCS on admission and ICH score were significantly associated with mortality.

Conclusion

After careful statistical review of the data obtained from this patient population, only low VEGF values were found to be a significant predictor of mortality. However, elevated CRP and Hcy levels were associated with a non-significant trend in hemorrhage size and mortality suggesting that CRP and Hcy-lowering therapies may decrease hemorrhagic stroke risk and severity.

## Introduction

Spontaneous intracerebral hemorrhage (ICH) accounts for 10-20% of all strokes and has a higher morbidity and mortality than ischemic stroke with 30-day mortality rates of 37 - 52% [1-2]. Medical management is the primary treatment modality and consists primarily of airway protection, oxygenation, tight blood pressure control, and correction of coagulopathy. Surgical intervention has limited efficacy compared to medical management and is often reserved for large hemorrhages causing mass effect or intraventricular hemorrhage (IVH) causing hydrocephalus. The pathogenesis of brain injury after intracerebral hemorrhage is thought to be due to mechanical damage followed by ischemic, cytotoxic, and inflammatory changes in the underlying and surrounding tissue [1-2]. Although the majority of research into the different biomarkers as predictors of stroke recurrence, severity, and outcome has been done with ischemic stroke, there is increasing research interest into the various inflammatory biomarkers and growth factors that are secreted during intracerebral hemorrhage. The biomarkers investigated in this study are tumor necrosis factor alpha (TNF alpha), C-reactive protein (CRP), homocysteine (Hcy), and vascular endothelial growth factor (VEGF). 

TNF alpha is a pro-inflammatory cytokine released via neuronal macrophages, microglial cells, and astrocytes during ischemic injury and plays a vital role in local inflammatory and thrombotic pathways [3]. TNF alpha has been found to be a predictor of acute ischemic stroke with 93.33% sensitivity and 96.75% specificity in the study by Tuttolomondo et al [3]. It has also been found to be a predictor of recurrent ischemic stroke likely due to its involvement in atherogenesis and atherothrombosis [4]. TNF alpha has also been correlated with infarct volume and early neurological deterioration in patients with levels > 14 pg/ml [5]. In ICH, high TNF alpha has been associated with the size of perihematomal edema, early hematoma growth which is associated with early neurologic deterioration, a poor functional outcome at three months, and increased mortality [2, 6].

C-reactive protein (CRP) is a pro-inflammatory cytokine that has been shown to be associated with worse outcome after ischemic stroke and an increased risk of recurrent stroke [4, 7]. Patients with higher CRP levels in acute ischemic stroke tend to have larger infarct volumes, and CRP levels > 1.5 mg/dl at discharge is a predictor of new vascular events (transient ischemic attack, cerebrovascular accident, myocardial infarction, unstable angina) and/or death at one year [8]. In ICH, CRP is associated with an increased 30-day mortality and added 8% improvement in the accuracy of the ICH score [9]. CRP levels > 5 mg/dL at admission and at 72 hours post-admission have worse Glasgow Outcome Score (GOS) and overall survival at six months [10].  

Homocysteine (Hcy) is an amino acid derived from methionine. Elevated levels are often due to a deficiency in B vitamins or genetic diseases involving mutations in cystathionine beta-synthase or the 5,10-methylenetetrahydrofolate reductase (MTHFR) gene [11]. Smoking, high methionine diet, and sedentary lifestyle are factors that contribute to elevated Hcy levels [11]. Hcy causes increased permeability of the blood-brain barrier and impairs the integrity of blood vessels and is a factor in the formation of atherosclerosis [11]. Elevated Hcy levels confer an increased risk of both ischemic and hemorrhagic stroke with no significant difference in Hcy levels [11-12]. Elevated levels also correlate with larger hematoma volume in the thalamic and basal ganglia ICH [13]. A level > 10.3 umol/L was an independent predictor of early neurologic deterioration in patients with ischemic stroke, and a level > 15 umol/L is an independent predictor of ischemic stroke [14-15]. Lowering Hcy with folate, vitamin B6, and B12 supplementation has been found to reduce the overall risk of stroke (ischemic and hemorrhagic) but not the severity of stroke [12, 16].

VEGF is a polypeptide growth factor that is a key mediator in angiogenesis and has been found to be elevated in patients with acute ischemic stroke. Higher concentrations of VEGF are found to be in the ischemic penumbra [17]. It is secreted via activated macrophages and microglial cells in response to hypoxic conditions in ischemic stroke and functions to increase vascular permeability, angiogenesis, and inhibit cell apoptosis [17-18]. In a rat study, injection of VEGF showed improved neurological recovery but increased blood-brain barrier leakage and increased hemorrhagic transformation [19]. VEGF is increased in ischemic stroke and has been found to be a predictor of poor functional outcome in patients with cardioembolic infarcts while being a predictor of good functional outcome in atherothrombotic infarcts [20]. One study of 95 patients with ICH showed elevated levels of VEGF to be associated with neurologic improvement and reduced residual cavity at three months post-hemorrhage [21]. In contrast, another study showed higher levels to be associated with worsening neurologic function at discharge [22]. The effects on the outcome of elevated VEGF levels in patients with intracerebral hemorrhage are unclear at this time based on the current literature.

The aim of this study was to investigate whether these biomarkers were abnormal in hemorrhagic stroke patients and if the effects of these biomarkers were a predictor of the clinical severity of ICH.

## Materials and methods

Study design

This was a retrospective review of a prospectively collected database of patients admitted to the neurosurgery service at Arrowhead Regional Medical Center from 2015 - 2017 with a diagnosis of ICH or IVH. In addition to the standard of care, patients had at least one of the following labs drawn: tumor necrosis factor alpha (TNF alpha), C-reactive protein (CRP), homocysteine (Hcy), and vascular endothelial growth factor (VEGF). Patients were included in the study if older than 18 years of age, had a diagnosis of spontaneous ICH or IVH without underlying trauma, vascular abnormality, or evidence of hemorrhagic transformation of ischemic stroke, and if they had at least one of previously mentioned biomarkers drawn on admission for ICH. A total of 42 patients were included in the study. We looked for a relationship between TNF alpha, CRP, Hcy, and VEGF with our primary outcomes, which included death, Glasgow Coma Scale (GCS) on admission, early neurologic decline (END), and hemorrhage size. Secondary outcomes included GCS on discharge, ICH score, functional outcome risk stratification scale of intracerebral hemorrhage (FUNC score), change in hemorrhage size, a need for surgical intervention, and the length of ICU (intensive care unit) stay.

All patients were admitted to the ICU for a minimum of 24 hours with neurological checks every hour. Patients were intubated at the discretion of the emergency department or if the GCS was ≤ 8. Systolic blood pressure (SBP) was strictly maintained with the goal of < 140 mmHg throughout the ICU Stroke Unit stay. An external ventricular drain (EVD) was placed under the following conditions: GCS was ≤ 8, extensive intraventricular involvement, symptomatic hydrocephalus, or a need for the use of intraventricular t-PA (tissue plasminogen activator) administration. Patients underwent bedside intraparenchymal hematoma evacuation and drain placement versus craniotomy or craniectomy depending on the size of hemorrhage, a midline shift, the patient’s clinical exam, and clinician discretion. Repeat computed tomography (CT) of the head was obtained six to eight hours from the initial CT scan. The volume of hemorrhages was calculated via the ABC/2 formula (A is the greatest hemorrhage in axial diameter, B is hemorrhage diameter at 90 degrees to the axial plane, and C is the number of image slices multiplied by slice thickness, all divided by 2) [23]. Hemorrhage volume was calculated for the initial and repeat CT scans by a single neurosurgery resident. Vascular studies were obtained if underlying vascular abnormalities were suspected. TNF alpha, CRP, Hcy, and VEGF levels were drawn on admission. The labs were performed by Quest Diagnostics. The following scoring systems were utilized to determine the patient's clinical status: ICH score (a predictor of 30-day mortality) and FUNC score (a predictor of functional independence at 90 days) [24-25]. An early neurologic decline was defined as a decrease in the GCS of two or more points within 48 hours of admission.

Statistical analysis

All statistical analyses were conducted using the SAS software for Windows version 9.3 (SAS Institute Inc., Cary, North Carolina). Descriptive statistics were presented as frequencies and proportions for categorical variables. Chi-squared, Mantel-Haenszel chi-square, and Pearson correlation coefficient were used in analyzing the data. All statistical tests were two-sided. P-value < 0.05 was considered to be statistically significant.

## Results

A total of 42 patients with spontaneous intracerebral hemorrhage and biomarkers were studied; of these, 12 patients (28.5%) required surgical intervention and four patients (9.5%) died. The different surgical procedures performed, along with the patient demographics, length of stay, and the average hemorrhage sizes, are shown in Table 1. Of the patients that died, three were admitted and presented with a GCS of 7T (Glasgow Coma Scale score of 7 with T indicating patient intubated) or less, three had an ICH volume > 30 cm^3^, two had lobar hemorrhages, and two had deep hemorrhages (basal ganglia with IVH). Three of the patients who died had elevated CRP levels ranging from 6.92 to 10.98 mg/dL, and one patient had an elevated Hcy level (13.2 umol/L). Compared to patients alive at discharge, the patients who died had a larger average initial ICH size, although this was not statistically significant (85.25 vs 15.75, p = 0.1658). One of the patients that died with an elevated CRP had a change in hemorrhage size of 13 cm^3^. There were 10 patients that lived with elevated CRP levels ranging from 1.51 to 43.23 mg/dL. Three patients underwent EVD placement, and one patient underwent decompressive craniectomy. Two patients underwent withdrawal of care by family, and both had an ICH volume of > 100 cm^3^. Nine patients had an ICH size > 30 cm^3^, and of those patients, the CRP was elevated in seven patients ranging from 6.41 - 10.98 mg/dL and the Hcy level was elevated in two patients ranging from 13.2 - 16.7 umol/ml. However, there were also six patients with an ICH size < 30 cm^3^ who had an elevated CRP level ranging from 1.51 to 43.23 mg/dL. There were also six patients with an ICH size < 30 cm^3^ with an elevated Hcy ranging from 11.9 to 21.7 umol/ml.

**Table 1:** Patient Demographics Demographics of patients, including gender, age, GCS on admission, ICU and hospital length of stay, number of procedures performed, and ICH sizes. GCS: Glasgow Coma Scale; ICH: intracerebral hemorrhage: ICU: intensive care unit

Demographics (n = 42)	
Male/Female	24/18
Age	58.5 ± 14.2
GCS on Admission	12.4 ± 3.8
ICU Length of Stay	5.6 ± 6.2
Hospital Length of Stay	10.5 ± 10.7
# of patients with procedures, each patient could have more than one procedure	12 (28.6%)
External Ventricular Drain	12
Intraparenchymal Drain	3
Ventriculoperitoneal Shunt	2
Craniectomy	2
ICH volume (cm^3^)	
Initial	22.4 ± 39.2
Repeat	23.6 ± 41.0
Change	1.2 ± 3.4
Midline Shift (mm) on initial ICH	2.8± 8.1
Mortality	4/42 deaths (9.5%)

CRP and Hcy levels were elevated in our study in 58% and 31% of patients tested, respectively (Table 2). With the exception of low VEGF serum values and mortality, when trending each of the biomarkers with our primary and secondary endpoints, higher values of the biomarkers did not show a significant correlation with any of the endpoints (Tables 3, 4). In our patients with ICH, TNF alpha, CRP, and Hcy levels were not found to predict mortality and were not correlated to GCS on admission or initial hemorrhage size. Only a low VEGF was significantly associated with death (Table 4). Although some patients with negative outcomes (including death) had elevated levels of CRP and Hcy, there were many patients with positive outcomes with elevated levels of the biomarkers, as well as a correlation between the biomarkers and ICH but not predicting outcomes. Also, when looking at secondary outcomes, TNF alpha, CRP, Hcy, and VEGF levels were not found to be correlated with GCS on discharge, ICH score, FUNC score, change in hemorrhage size, and length of ICU stay. These biomarkers were also not able to predict the need for a surgical procedure.

**Table 2:** Average Values of Biomarkers in Patients Upon Arrival CRP: C-reactive protein; ICH: intracerebral hemorrhage; SD: standard deviation; TNF alpha: tumor necrosis factor alpha; VEGF: vascular endothelial growth factor

Biomarkers and percent elevated in ICH	Percent Elevated	Study Average + SD	Institutional Normal Values
Homocysteine (Hcy) (umol/L) (n = 26)	31%	12.3 ± 7.0	< 11.4 umol/L
TNF alpha (pg/mL) (n = 24)	42%	1.3 ± 0.8	0.56 - 1.40 pg/ml
CRP (mg/dL) (n = 36)	58%	3.9 ± 7.8	< 0.50 mg/dL
VEGF (pg/mL) (n = 27)	15% (56% low normal)	54.8 ± 37.6	31 - 86 pg/ml

**Table 3:** Correlation of Biomarkers to Various Endpoints Using the Pearson Correlation Coefficient The Pearson correlation coefficient, r, can take a range of values from +1 to -1. A value of 0 indicates that there is no association between the two variables. A value different than 0 indicates an association; that is, as the value of one variable changes, so does the value of the other variable. Coefficient value strength of association is as follows: 0.1 < | r | < .3 small correlation, 0.3 < | r | < .5 medium or moderate correlation, | r | > .5 large or strong correlation. CRP: C-reactive protein; FUNC: functional outcome risk stratification scale of intracerebral hemorrhage; GCS: Glasgow Coma Scale; ICH: intracerebral hemorrhage; ICU LOS: intensive care unit length of stay; TNF alpha: tumor necrosis factor alpha; VEGF: vascular endothelial growth factor

	ICH Score	ICU-LOS	FUNC	Admit GCS	Midline Shift	Initial ICH Size	Change in ICH Size
Homocysteine (Hcy)	0.038 (p=0.85)	-0.054 (p=0.79)	-0.050 (p=0.8)	-0.188 (p=0.35)	0.129 (p=0.52)	0.021 (p=.92)	0.311 (p=0.12)
TNF alpha	-0.171 (p=0.43)	-0.020 (p=0.92)	0.029 (p=0.89)	0.158 (p=0.46)	-0.122 (p=0.57)	-0.195 (p=0.36)	-0.049 (p=0.82)
CRP	0.168 (p=0.33)	0.153 (p=0.37)	-0.252 (p=0.13)	-0.089 (p=0.61)	0.135 (p=0.44)	0.143 (p=0.41)	0.117 (p=0.49)
VEGF	-0.125 (p=0.53)	0.108 (p=0.59)	-0.032 (p=0.87)	-0.186 (p=0.35)	0.009 (p=0.96)	-0.024 (p=0.91)	-0.123 (p=0.55)

**Table 4:** Analysis of Factors Associated with Mortality * Indicates factors that are statistically significant predictors of mortality CRP: C-reactive protein; GCS: Glasgow Coma Scale; ICH: intracerebral hemorrhage: n: number; TNF alpha: tumor necrosis factor alpha; VEGF: vascular endothelial growth factor

Factors	Alive (n = 38)	Death (n = 4)	P-value
Age	58.18 ± 14.22	61.25 ± 15.73	0.6863
GCS on admission	12.97 ± 3.25	7.25 ± 4.79	*0.0026
Initial ICH Size (cm^3^)	15.75 ± 27.51	85.25 ± 76.33	0.1658
Repeat ICH Size (cm^3^)	16.51 ± 28.67	91.25 ± 78.27	0.1516
Change in ICH Size (cm^3^)	0.74 ± 2.54	6 ± 6.48	0.2026
TNF alpha (pg/ml)	1.32 ± 0.83	1.07 ± 0.54	0.6798
Homocysteine (Hcy) (umol/L)	12.3 ± 7.28	12.15 ± 1.48	0.9774
CRP (mg/dL)	3.51 ± 8.05	8.41 ± 2.24	0.3067
VEGF (pg/mL)	56.68 ± 38.51	31 ± 0	*0.0028
Midline Shift	0.95 ± 2.79	10.75 ± 13.25	0.2355
ICH Score on admission (30 day mortality)			*0.0036
0 (0%)	12 (31.6%)	0 (0%)	
1 (13%)	20 (52.6%)	0 (0%)	
2 (26%)	1 (2.6%)	0 (0%)	
3 (72%)	2 (5.3%)	1 (25%)	
4 (97%)	3 (7.9%)	3 (75%)	
Early Neurologic Decline			0.2676
No	35 (92.1%)	3 (75%)	
Yes	3 (7.9%)	1 (25%)	

An early neurologic decline occurred in four patients (9.5%) with one death in this group. This was not significantly associated with mortality (Table 4). However, the patient that died had an elevated CRP level of 10.98 mg/dL. Of the patients with an early neurological decline, two out of two patients with TNF alpha labs drawn had elevated levels. The CRP level was elevated in two out of three patients that had labs drawn. Hcy was elevated in one out of three patients with labs drawn. Higher ICH score and GCS on admission, while not statistically associated with biomarkers, were found to be associated with mortality (Table 4). 

Of the patients who returned to the hospital within six months after discharge, two had elevated Hcy levels and three had elevated CRP levels. None of these patients experienced a repeat stroke of any kind. The remainder of the patients who had elevated levels were not seen at the hospital after discharge.

## Discussion

Our study only demonstrated a statistically significant association between low VEGF serum value and death. It did not demonstrate a statistical significance between these four biomarkers and other primary and secondary outcomes in hemorrhagic stroke. Although our study did not demonstrate an association, many others have done so (as seen in Table 5), giving rise to the belief that these markers could be used to modify the management of patients with any kind of stroke [2-15, 17, 19-22]. Of the four markers, CRP and Hcy levels were elevated in 58% and 31% of patients tested, respectively. Further review of the patients that died revealed a trend of increased mortality and larger ICH size in patients with elevated CRP and Hcy levels, although this specific correlation was not statistically significant. The lack of significance may be attributed to the small number of patients with the available biomarkers for study and the need to further refine an association, such as the location and cause of ICH, with outcomes.

**Table 5:** Comparison of Biomarkers in Literature Comparison of literature associations of various inflammatory markers between ischemic and hemorrhagic stroke. *CRP and Hcy are elevated in our patients. CRP: C-reactive protein; ICH: intracerebral hemorrhage; TNF alpha: tumor necrosis factor alpha; VEGF: vascular endothelial growth factor

	Ischemic Stroke	Hemorrhagic Stroke
TNF alpha	93.33% sensitivity and 96.75% specificity in predicting stroke [3]. Increased risk of recurrent stroke [4]. Increased infarct volume and early neurologic deterioration [5].	In ICH, TNF alpha has been associated with the size of the perihematoma edema, early hematoma growth which is associated with early neurologic deterioration, poor functional outcome at three months, and increased mortality [2, 6].
CRP*	Worse outcome and increased risk of recurrent stroke [4, 7]; larger infarct and new vascular events [8].	Increased 30-day mortality and improvement in ICH score predictive value [9]; worse Glasgow outcome score and worse overall six-month survival [10].
Homocysteine* (Hcy)	Increased risk of ischemic stroke [11-12]; a predictor of early neurologic decline and ischemic stroke [14-15].	Increased risk of hemorrhagic stroke [11-12]; larger hematoma volume (thalamic, basal ganglia) [13].
VEGF	Elevated in ischemic penumbra [17]; increased recovery but also increased hemorrhagic conversion in a rat study [19]; poor functional outcome in cardioembolic stroke but good functional outcome in atherothrombotic stroke [20].	Elevated levels associated with neurologic improvement and reduced residual cavity [21]; another study found elevated VEGF to be associated with worse neurologic function at discharge [22].

These four biomarkers are abnormal in patients with hemorrhagic stroke. However, more high powered studies are needed to determine whether there is a significant predictive value in ICH patients. The lack of significance in our study does not exclude the positive correlation between these biomarkers and mortality and the ICH size. Additionally, there were patients with CRP and Hcy levels that were higher than the patients that died; these patients did not have early neurologic decline nor any further complications. Of the patients that died in our study, three patients had elevated CRP levels ranging from 6.92 to 10.98 mg/dL and the fourth patient had an elevated Hcy level of 13.2 umol/L. One of the patients that died with an elevated CRP had a change in hemorrhage size of 13 cm^3^. The patients with elevated CRP had ICH sizes of 31.5 cm^3^, 38 cm^3^, and 183 cm^3^, and the patient with an elevated Hcy level had an ICH size of 107 cm^3^.

The GCS and ICH score on admission, as well as the low VEGF, were all significantly associated with higher mortality in our patients. Hemorrhage size, although not found to be statistically significant with mortality in our study, is an established risk factor for mortality in ICH [25]. Nine patients had an ICH size > 30 cm^3^, the CRP was elevated in seven patients (ranging from 6.41 - 10.98 mg/dL), and the Hcy level was elevated in two patients (ranging from 13.2 - 16.7 umol/ml). This further shows a non-statistically significant trend of increased CRP and Hcy levels being associated with a larger hemorrhage size.

An additional consideration is in the elevation of biomarkers in patients with early neurological decline. In our study, all patients who experienced early neurological decline had elevated biomarkers of TNF alpha (100%), CRP (67%), and Hcy (33%). This leads us to believe that there is a possible correlation of TNF alpha and CRP with early neurological decline; a stronger connection could be delineated when comparing both TNF alpha and CRP in conjunction with one another as the one patient who had both labs drawn showed a significant elevation in both biomarkers. More patients with early neurological decline would need both labs drawn to further investigate this apparent connection.

Of the markers tested in this study, Hcy, as well as CRP, can be affected by lifestyle and dietary modifications. The elevation of CRP further promotes the idea that an inflammatory process occurs after hemorrhagic stroke [1-2]. By showing this elevated level, we give rise to the possibility of CRP-lowering therapy in high-risk individuals. Exercise, weight loss, and diet have all been successful in lowering CRP levels [26]. Medications that were commonly taken for hypertension (angiotensin-converting enzyme inhibitors/angiotensin receptor blockers), high cholesterol (statins), diabetes, inflammation (cyclooxygenase inhibitors), and antiplatelet agents (clopidogrel, abciximab) have also been found to lower CRP levels [27]. Vitamin C supplementation may be another potential treatment modality as it is decreased in patients with ICH and supplementation has reduced CRP levels in patients with cardiovascular disease [28-29]. Studies regarding vitamin C supplementation and ICH are lacking. Patients at increased risk should be encouraged to pursue these lifestyle changes, along with the appropriate medical therapy, to reduce their overall risk of hemorrhagic stroke.

Multiple studies show elevated levels of Hcy convey an increased risk of ischemic and hemorrhagic stroke, as well as an increased risk of recurrent stroke [11-12, 16]. Although none of the patients with elevated Hcy or CRP that returned to the hospital had a recurrent stroke, the number of patients was too small to draw a conclusion and should not negate the potential benefits of lowering these levels in patients. Smoking cessation, regular exercise, and a low methionine diet may help lower Hcy levels and therefore decrease the risk of stroke [11-12]. A daily combination therapy of 2.5 mg folic acid, 50 mg vitamin B6, and 1 mg of vitamin B12 has been shown to decrease the risk of overall stroke (hemorrhagic and ischemic) in patients with known cardiovascular disease [13]. Our findings, along with evidence in the literature, justifies the recommendation of Hcy testing in high-risk individuals, such as those with co-morbidities or previous occurrences of either ischemic or hemorrhagic stroke. In individuals with elevated Hcy levels, as in our study, the administration of Hcy-lowering therapy could potentially decrease the risk of hemorrhagic stroke.

Previous studies show that VEGF has neuroprotective effects and promotes neurogenesis [21]. This suggests that high VEGF levels could favor a better prognosis. In our study, two of the patients who died had VEGF labs drawn, with values of 31 pg/ml each. This is on the lowest end of our institution's normal values of 31 - 86 pg/ml. Our study found an association with low VEGF levels and mortality which further supports VEGF being neuroprotective. However, some patients with very low VEGF levels also did well, which leads us to believe that outcome after ICH is multifactorial. Further investigations regarding VEGF and ICH outcome is warranted, as well as increasing VEGF levels as a potential treatment modality. However, utilizing what we know about this biomarker can better identify those who are at higher risk for worse outcomes if it is drawn on presentation.

The biggest limitation of this study was the lack of uniformity in our patient population in regards to the biomarkers that were drawn. Only 24 patients had TNF alpha levels, 26 had Hcy levels, 36 had CRP levels, and 27 had VEGF levels. As these labs are not the standard of care when managing patients with hemorrhagic stroke, many patients either have labs canceled or not ordered altogether. This is also associated with another limitation - the lack of a specified control group. Although baseline levels are obtained in healthy subjects and in ischemic stroke patients from many other studies, these values vary greatly from study to study. Additionally, there was no routine follow-up with the patients in this study, making associations with long-term morbidity and mortality not possible. However, no patients had a re-bleed during the hospitalization. Further inspection of the patient population shows many co-morbidities, such as hypertension (32/42), diabetes (14/42), and illicit drug use (8/42), in many of the patients. These factors could confound the results of both the severity of the stroke and the change in serum levels of the studied biomarkers. These factors need to be individually studied as well in order to reduce the effect of confounding.

## Conclusions

After careful statistical review of the data obtained from this patient population, only low VEGF levels were associated with mortality. However, CRP and Hcy levels were elevated in 58% and 31% of the patients tested, respectively. There was also a non-significant trend towards increased hemorrhage size and mortality with elevated CRP and Hcy levels. This would suggest that CRP and Hcy-lowering therapy could reduce the risk and/or severity of hemorrhagic stroke. These markers could potentially be followed and treated in patients with prior stroke or patients at risk of stroke in order to potentially decrease their risk of hemorrhagic stroke.
